# The First Cleaner Ant? A Novel Partnership in the Arizona Desert

**DOI:** 10.1002/ece3.73308

**Published:** 2026-04-12

**Authors:** Mark W. Moffett

**Affiliations:** ^1^ Department of Entomology National Museum of Natural History Washington DC USA

**Keywords:** cleaner fish, harvester ant, mutualism, pheromones, symbiosis

## Abstract

I give an account of the first known example of an ant (i.e., an undescribed *Dorymyrmex*) that licks and nips the much larger workers of a different ant species ( *Pogonomyrmex barbatus* ) in a manner remarkably parallel to the actions of cleaner fish that clean other species of fish. Specifically, the potentially aggressive individuals being tended encourage these attentions by stationing themselves in a distinctive, rigid posture at particular locations (in the case of the ant, near the nest of the cleaner species) and even permit a cleaner to inspect between their open mandibles. The payoffs of this activity for both the cleaners and the tended workers have yet to be worked out.

## Graphical abstract

I give an account of an undescribed ant species from Arizona that licks and nips the much larger workers of a different ant species in manner remarkably parallel to the actions of cleaner fish that clean other kinds of fish. Specifically, the potentially aggressive individuals being tended encourage these attentions by stationing themselves in a distinctive, rigid posture at particular locations (in the case of the ant, near the nest of the cleaner species) and will even permit a cleaner to inspect between their open mandibles.

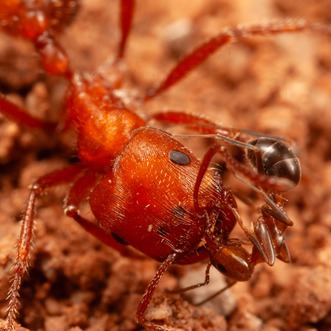

On the first morning of a five‐day visit in mid‐June to the Southwestern Research Station in Portal, Arizona, I was behind my residence at Quailway Cottage (152 Portal Rd., 31.879, −109.060), watching *Pogonomyrmex barbatus* harvester ants spill from their nests in the mesquite desert, when I noticed something odd. A few scattered “Pogos,” as myrmecologists call them, seemed frozen in place. Walking all over these curiously statue‐still workers were smaller *Dorymyrmex*, or cone ants, later identified by Stefan Cover at Harvard as an undescribed species close to *D*. *medeis*. I soon saw that each Pogo would stand rigidly until a “Dory” arrived, then hold that pose as the visitor explored her body surface (note that all worker ants are female). I watched at least ninety such inspections over the following days.

The year was 2006. I had hoped to return to gather additional hard data for insights on the function of this remarkable behavior. I never did, but over the course of those days I captured images documenting each step of these interactions, some of which I publish here for the first time.

The activity started at sunrise and peaked before 9:00 a.m., when the ants would retreat in the heat of the day. The cone ants had multiple unadorned nest entrances around the cleared “pan” encircling each Pogo entrance in an area extending wide enough that doubtless multiple colonies of both species were involved. An occasional Pogo came within 5 cm of a Dory entrance to adopt a distinctive posture, standing stiffly high on her legs with her gaster typically lifted and mandibles agape. Usually within the minute a cone ant would climb onto the harvester ant, licking (Figure 1) and nipping or pulling (Figure 2).

**FIGURE 1:**
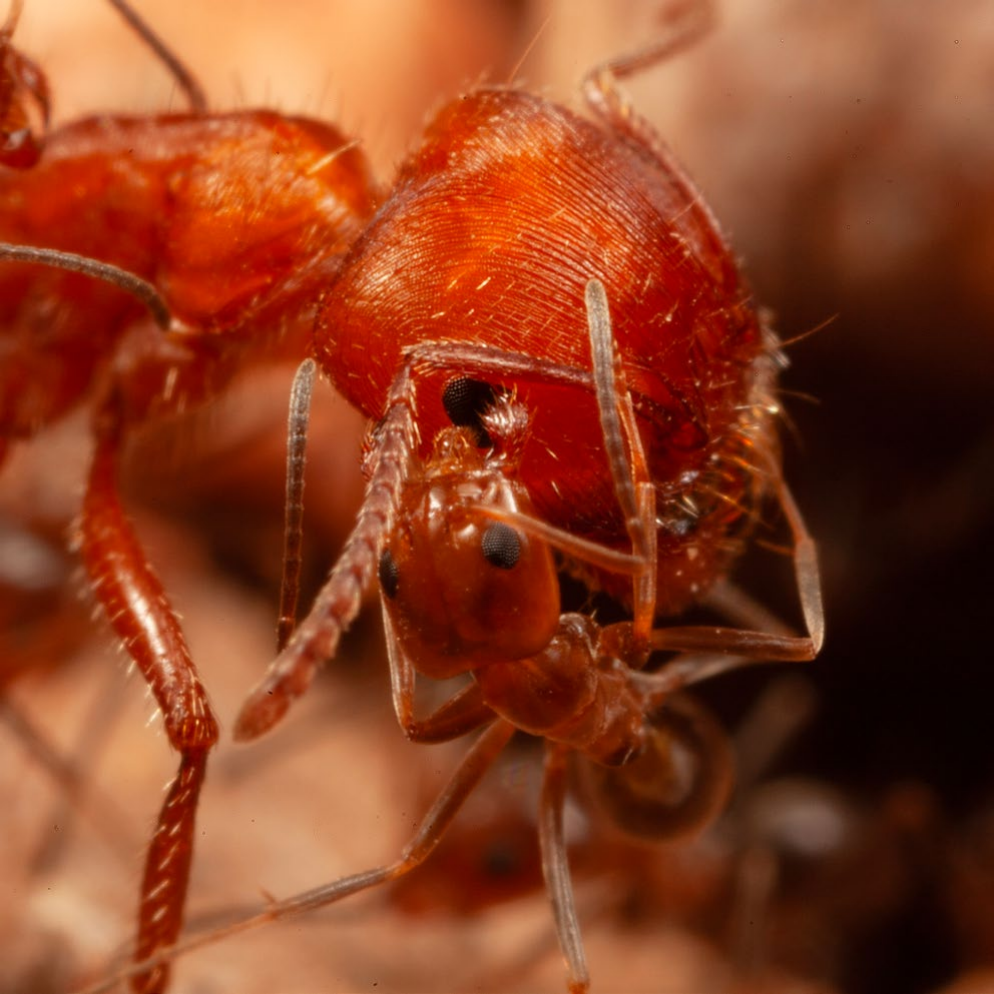
A cone ant worker licking near the eye of a *Pogonomyrmex barbatus* worker.

**FIGURE 2:**
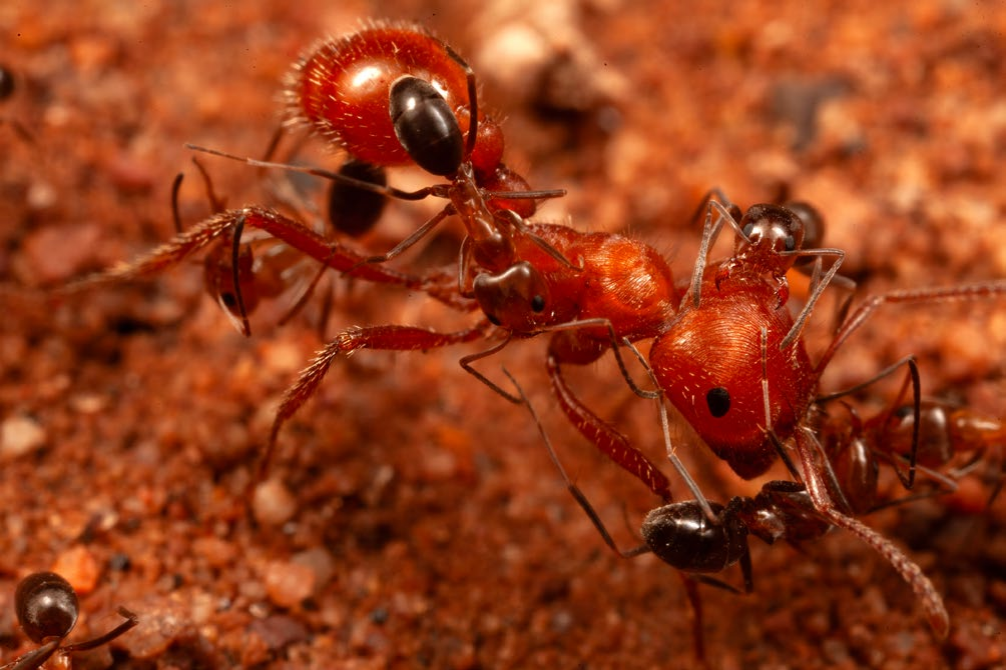
Five cone ant workers exploring the body surface of a *Pogonomyrmex barbatus* .

The Pogo tolerated this attention for at least a few seconds, never biting back. In a sample of 32 interactions watched from the beginning, four lasted less than 15 s, 13 were under 1 min, and two continued beyond 5 min; over time, as many as five cone ants could accumulate on one Pogo (Figure 2). The restraint of the Pogos was clear in three instances when pairs of workers that happened to be adjacent to each other stayed immobile while a cone ant wandered from one to the other (Figure 3). Only living Pogos elicited these attentions; Pogos I had killed in a freezer and then thawed were examined by cone ants but not treated the same way.

**FIGURE 3:**
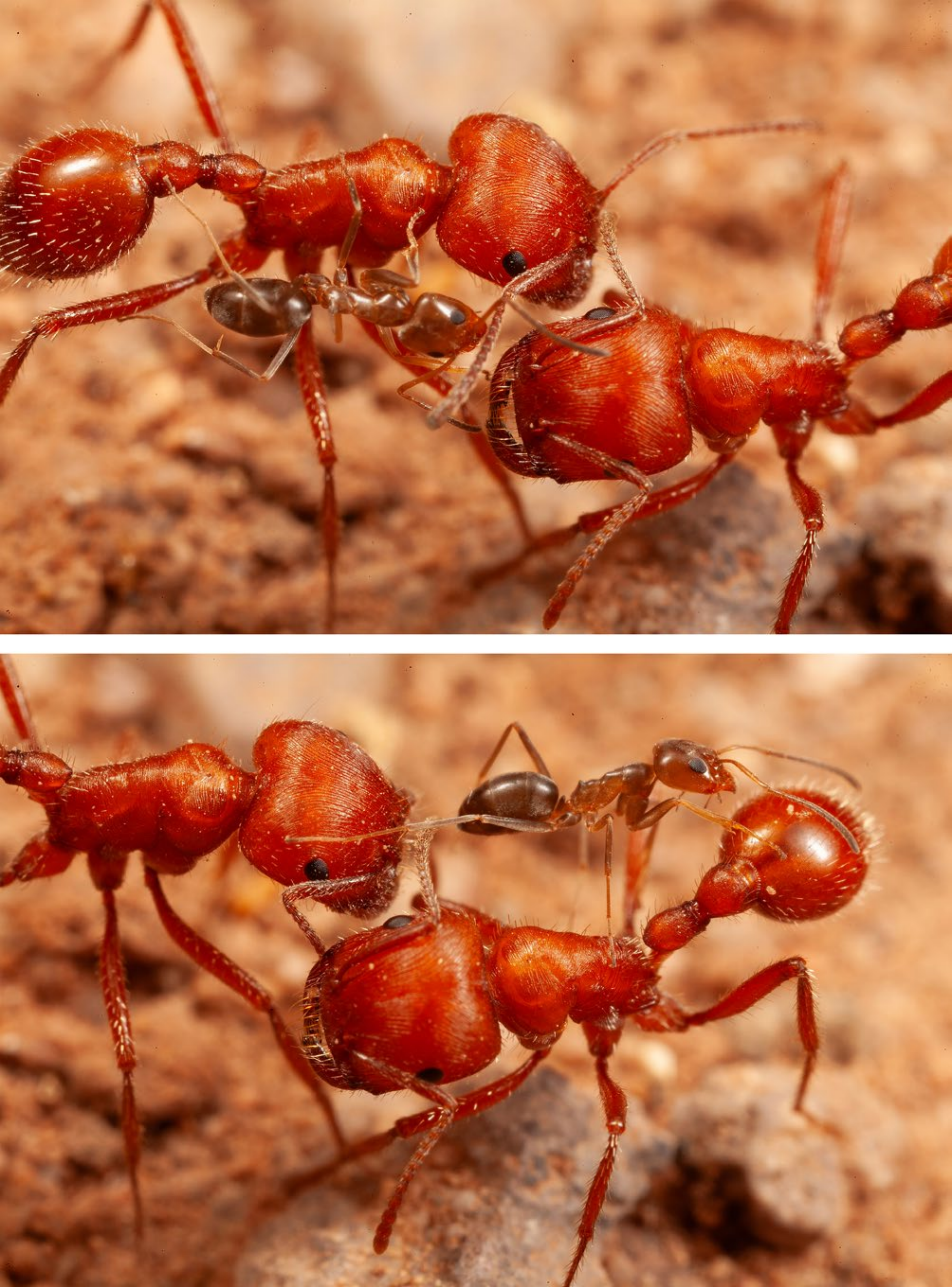
A cone ant walking from one stationary *Pogonomyrmex barbatus* worker to another.

Were the cone ants burgling morsels from the mouths of another species, as some ants do? Christina Kwapich of University of Central Florida tells me that a Florida *Forelius* poaches food from another harvester ant species, *Pogonomyrmex badius* , after which the robbed ant stiffens briefly but fails to defend herself. As far as I could detect, none of the Arizonan Pogos had been holding anything in their mandibles, and the cone ants were not attempting to elicit food exchange (i.e., trophallaxis, which Pogos do not do). Hence they were not stealing from their cousins. But two fascinating behaviors stood out. Several times I saw a Pogo back up directly into a cone ant nest entrance, where she waited until a worker from the colony climbed aboard (Figure 4). More remarkable still, the cone ants would lick and nibble *between the mandibles* of the larger Pogo (Figure 5). I was reminded in the first instance of how marine fish seek out special “stations” occupied by cleaner fish and, in the second, of how cleaner fish (or shrimp) will even feed inside the gaping jaws of a more massive, potentially dangerous, predatory fish. As I watched the ants interact, I became increasingly convinced that this *Dorymyrmex* was the first recorded “cleaner ant.”

**FIGURE 4:**
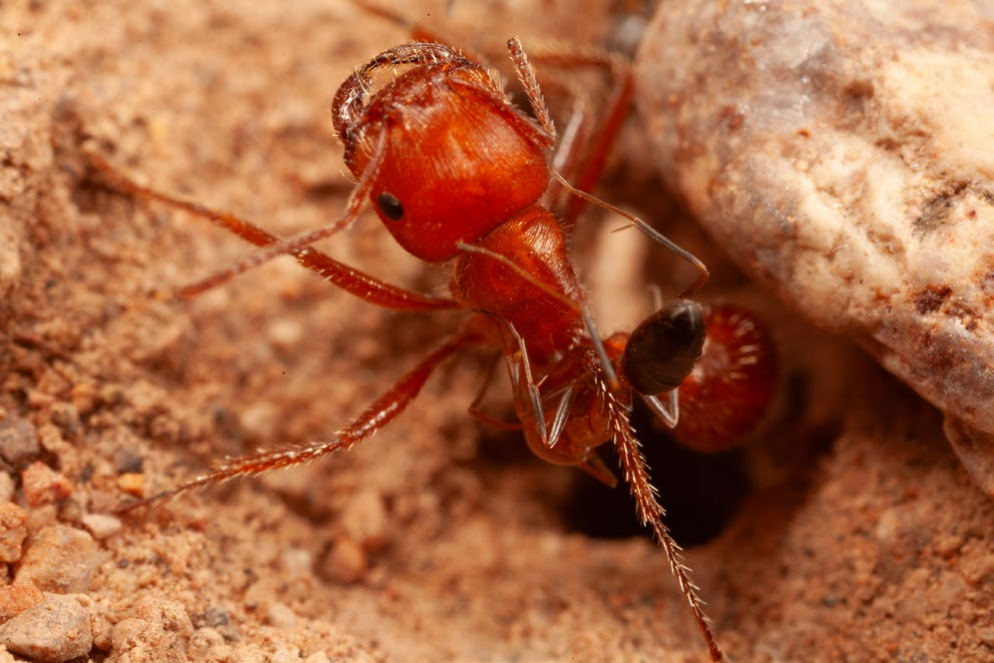
A *Pogonomyrmex barbatus* worker that has backed into a cone ant nest entrance to encourage cleaning by one of its workers.

**FIGURE 5:**
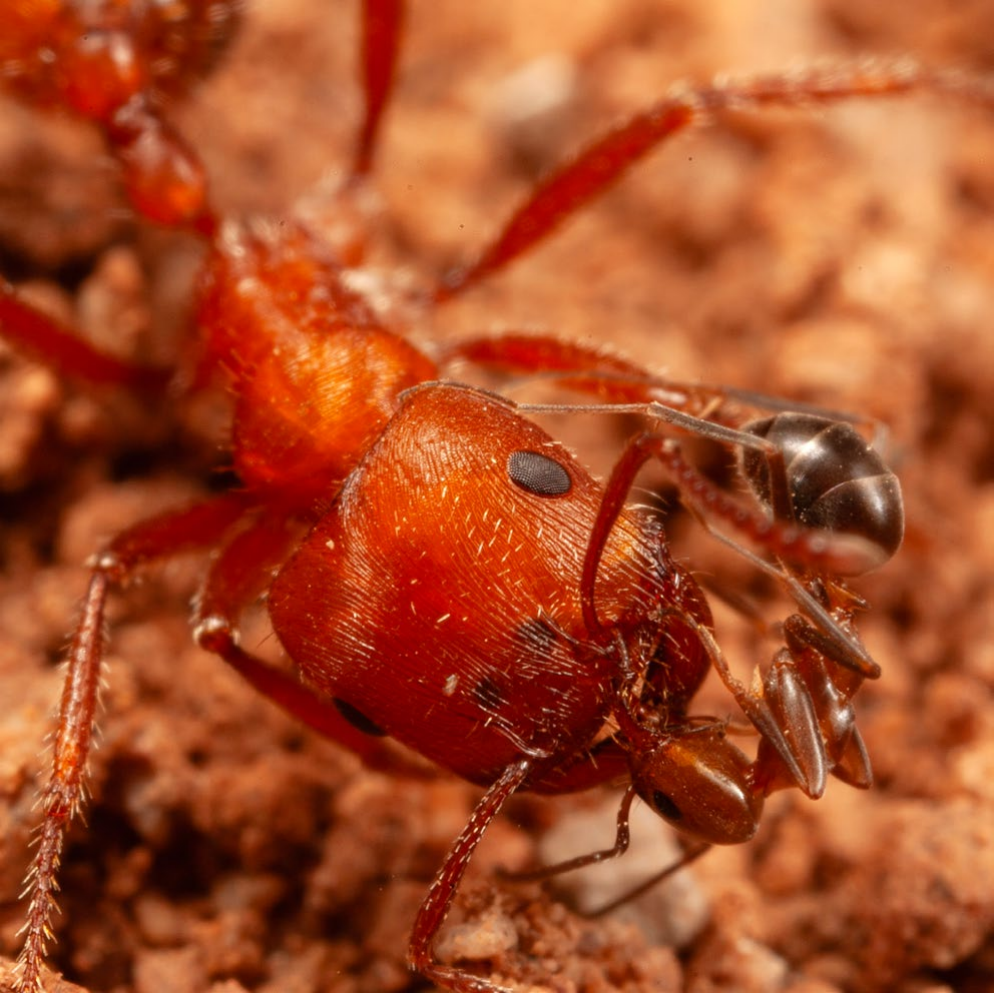
A cone ant exploring between the mandibles of a *Pogonomyrmex barbatus* worker.

As with cleaner fish, a bout generally ended when the cone ant appeared to annoy the Pogo: perhaps one bit too hard, or too many had climbed aboard, or the client ant had received a sufficient scrubbing. The cone ants could be jolted off so violently that the Pogo landed upside down (Figure 6). Any remaining passengers were knocked to the side as she righted herself and hurried away.

**FIGURE 6:**
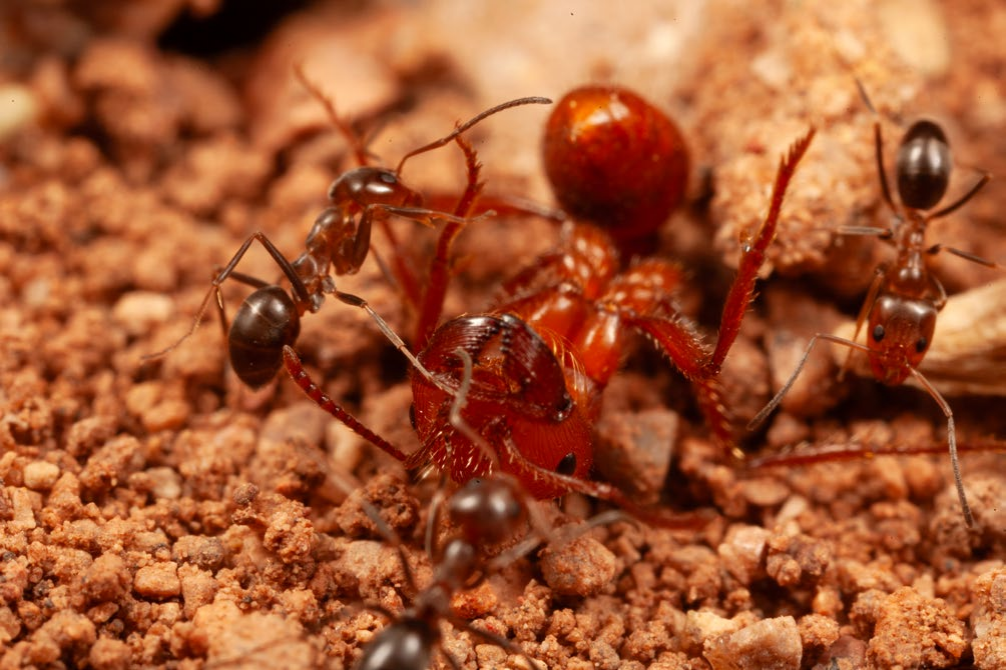
A *Pogonomyrmex barbatus* worker that has thrown herself onto her back to remove two cone ants after an extended period of being cleaned by them.

Harvester ants groom one another in their nest, potentially removing debris, contaminants, dead tissue, spores, and ectoparasites. What then could be the advantage of adding an interspecies assistant? The tended workers looked healthy and clean; I saw no ectoparasites that the cleaners might extract, and though a few Pogos had missing limbs, the wounds did not appear to be the focus of their efforts. Likely the cone ants access tighter corners than other Pogos could, possibly removing hard‐to‐reach pathogens; or maybe the *Pogonomyrmex barbatus* at Portal has outsourced the labor of grooming to another species; or, as University of Queensland marine biologist Alexandra Grutter hypothesizes, the ants are exchanging valuable microbes, creating a healthier microbiome for the cone ants or perhaps both species. Auburn University professor Clint Penick has found that at least one *Dorymyrmex* species produces antifungal and antibacterial substances that could conceivably be transferred to the *Pogonomyrmex badius* , which lacked such compounds when tested alongside *Dorymyrmex*. Adria LeBoeuf of the University of Cambridge proposed to me a nutritional payoff for the cone ants: a Pogo is likely to be lipid rich from eating seeds, any compounds on the integument ingested by cone ants included—another possible “byproduct benefit” in which cooperation arose as a fortuitous consequence of inherently selfish actions. Howard Topoff at the City University of New York points out that whatever else the cone ants gain, extended contact between the species could lead to the transfer of colony pheromones that might pacify the pugnacious Pogos, making it easier for the cone ants to interact with them and even nest nearby.

My uncertainties regarding whether the interactions are mutually advantageous, and what each participant provides to yield those advantages to the other, which slowed me from publishing this report, turn out to apply widely across presumptive “cleaner” organisms. For example, while client fish by all appearances seek out inspections and submit freely to them (the two criteria, met by the ants, that are usually treated as sufficient for calling the attending species a “cleaner fish”), in most cases a central question remains as to whether the relationship is mutualistic—whether being tended by a particular kind of fish not only significantly removes harmful substances or organisms but boosts the recipient's health. In fact, while over 200 species in 108 genera have been described as cleaner fish, studies showing an improvement in client survivorship have focused almost entirely on one wrasse, *Labroides dimidiatus* ; as for the shrimp, even though many species in several families have been known for many decades to climb onto fish, evidence of what they are actually removing with their pincer‐like claws has been spotty at best until relatively recently.

What of future research on ants? *Pogonomyrmex* could be coated with a fluorescent dye to see whether it shows up in the crops of tending *Dorymyrmex*. Determining what Pogos gain from encouraging the Dorys will take some doing. Comparing the general health and the symbiotic microbial communities of harvester ants that have access to cone ants with those that don't may require the prolonged removal of the cone ant population around some of their colonies (after determining whether Pogos elicit cleaning only near their nests and not farther afield). Metagenomic sequencing would clarify any differences in the microbes on the body surfaces of the individual workers that have undergone cleaning or for those colonies associated with cone ant nests.

The closest known parallel to this cleaning behavior among the insects might be found in some of the inquilines, or “guests,” that live with social species, such as the beetles studied by Joseph Parker at CalTech and his colleagues that rub the ants with their tarsi, or the “ant crickets” that lick their hosts in much the same way that the ants groom each other, in both cases likely transferring ant‐colony‐identifying hydrocarbon pheromones onto their own bodies. The early Harvard myrmecologist William Morton Wheeler called the *Myrmecophila* crickets “strigilators,” after the strigil tool the ancient Greeks devised to scrub themselves while bathing. While such activities have been described as “grooming,” a word choice that implies a benefit to the recipient, at least in terms of a significant cleaning, these relationships are generally considered exploitative, as the crickets, for example, eat the foraged food and sometimes the eggs of the ants. Still, the possibility that their attentions remove deleterious microbes, or aid in spreading advantageous ones, remains to be explored.

Nondescript as *Dorymyrmex* seem, they turn out to be hard to match for their behavior; the genus includes not just cleaners but *D. bicolor* (for a time placed in *Conomyrma*, as was *medeis*), which drops pebbles on competitors emerging from their nests to keep them from foraging. And cleaner behavior might not be unique to *Dorymyrmex*: University of Florida biologist Elizabeth Cash has a three minute video of *Forelius* workers (sister genus to *Dorymyrmex*) in Arizona briskly running over, and apparently occasionally grooming, a worker of the same Pogo harvester ant species.

In a world where most ants steal, fight, and outmaneuver one another, it seems that two species in an Arizona desert leave room for an unexpected collaboration, at cleaning stations where the behavior of the humble pismire once again delights and surprises.

## Author Contributions

**Mark W. Moffett:** conceptualization (equal), data curation (equal), formal analysis (equal), funding acquisition (equal), investigation (equal), methodology (equal).

## Funding

This work was supported by the John Templeton Foundation, 61819.

## Conflicts of Interest

The author declares no conflicts of interest.

## Data Availability

In this a descriptive article, all data are contained within the manuscript.
